# FPCB as an Acoustic Matching Layer for 1D Linear Ultrasound Transducer Arrays

**DOI:** 10.3390/s22155557

**Published:** 2022-07-25

**Authors:** Taemin Lee, Joontaek Jung, Sang-Mok Lee, Jongcheol Park, Jae-Hyeong Park, Kyung-Wook Paik, Hyunjoo J. Lee

**Affiliations:** 1 School of Electrical Engineering, Korea Advanced Institute of Science and Technology, Daejeon 34141, Korea; leetm@kaist.ac.kr (T.L.); mock0920@kaist.ac.kr (S.-M.L.); 2 Office of Nano Convergence Technology, National NanoFab Center, Daejeon 34141, Korea; jjt@nnfc.re.kr (J.J.); jcpark@nnfc.re.kr (J.P.); 3 Samsung Foundry, Samsung Electronics Co., Ltd., Hwaseong 18448, Korea; jh3375.park@samsung.com; 4 Department of Materials Science and Engineering, Korea Advanced Institute of Science and Technology, Daejeon 34141, Korea; kwpaik@kaist.ac.kr

**Keywords:** FPCB, composite, ACF, ultrasound transducer, acoustic matching layer, acoustic impedance

## Abstract

An acoustic matching layer is an essential component of an ultrasound transducer to achieve maximum ultrasound transmission efficiency. Here, we develop a flexible printed circuit board (FPCB) with a composite structure consisting of multiple polyimide and copper layers and demonstrate it as a novel acoustic matching layer. With a flexible substrate and robust ACF bonding, the FPCB not only serves as an acoustic matching layer between piezoelectric elements and the surrounding medium but also as a ground for the electrical connection between the transducer array elements and the folded substrate. A 1D linear ultrasound transducer array with the FPCB matching layer exhibits larger output pressure, wider -3dB bandwidth, and higher ultrasound beam intensity compared to that of an ultrasound transducer array with the alumina/epoxy matching layer, which is one of the most commonly applied composite matching layers. The enhanced transmission performance verifies that the proposed FPCB is an excellent matching layer for 1D linear ultrasound transducer arrays.

## 1. Introduction

Ultrasound transducers, typically composed of a piezoelectric element with matching and backing layers, convert electrical energy into mechanical energy and vice versa [1]. For the efficient delivery of acoustic energy from the transducer to the medium, the difference in acoustic impedances between the transducer and the transmission medium must be minimized. However, widely used piezoelectric materials such as lead zirconate titanate (PZT), lead metaniobate, and modified lead titanates exhibit relatively high acoustic impedances (20–30 MRayl) compared to that of human tissue (1.5 MRayl) [2]. Due to this large mismatch in acoustic impedance, it is inappropriate to use the piezoelectric material without a matching layer in biomedical applications. An acoustic matching layer, whose acoustic impedance is lower than the piezoelectric elements and higher than human tissue, needs to be placed at the interface to minimize the ultrasound reflection and maximize the ultrasound transmission efficiency. Thus, designing an optimal acoustic matching layer for ultrasound applications is critical to maximizing ultrasound transducer performance such as intensity and frequency bandwidth.

One of the traditional acoustic matching techniques is the quarter wavelength matching method, which requires the total thickness of the matching layer to be equal to the quarter wavelength of the ultrasound and acoustic impedance to be the geometric mean of the acoustic impedance of the piezoelectric element and the transmission medium [3]. Using a homogeneous material as an acoustic matching layer enables precise thickness control. However, a single-phase material that satisfies the acoustic impedance for biomedical applications (i.e., matching between human and piezoelectric materials) is not available. To overcome this challenge, composite films that are composed of high impedance metal particles and low impedance polymers or epoxy resins, such as silver/epoxy composites [4], alumina/epoxy composite film [5], and tungsten/epoxy composite film [6,7], have been developed and applied as acoustic matching layers for ultrasound transducers. The desired acoustic impedance of the matching layer can be obtained by varying the volume fraction of metal powders and polymers. However, the large particle size of the powders and inconsistent mixing cause non-uniform film thickness, high surface roughness, and noticeable variation in the acoustic impedance of the film [8,9]. Moreover, for higher ultrasound frequency applications, it is more challenging to control the layer thickness via the lapping process [10]. In addition, the fabrication of the composite matching materials requires the repetition of time-consuming processes followed by mixing metal powders with epoxy resin, spin-coating, and curing to achieve the desired thickness of the material [11].

**Figure 1:**
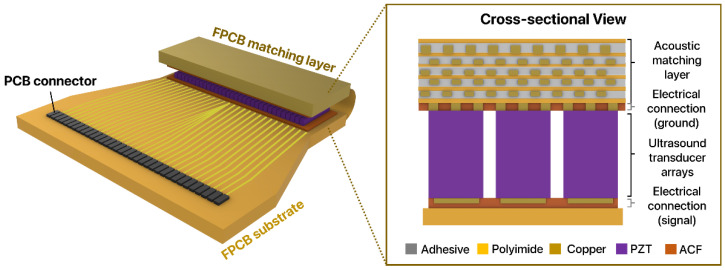
Conceptual diagram of a FPCB substrate developed as a matching layer for a 1D linear ultrasound transducer array.

In this work, we propose a novel acoustic matching technique by using a flexible printed circuit board (FPCB) as an acoustic matching layer for 1D linear ultrasound transducer arrays (Figure 1). The proposed acoustic matching layer is a multi-layered composite structure composed of alternating polyimide (PI) and copper (Cu) layers. The acoustic impedance of the matching layer can be readily controlled by varying the volume fraction of copper and polyimide. By folding the FPCB substrate and bonding it to another substrate via anisotropic conductive film (ACF), the FPCB not only serves as an acoustic matching layer between piezoelectric elements and the surrounding medium but also as a ground for the electrical connection between the transducer array elements and the folded substrate. Since FPCBs are widely used substrates for electrical packaging, using the same FPCB with varying volume fractions is a low-cost yet elegant means to provide matching for ultrasound biomedical applications. Here, to evaluate whether the proposed FPCB is a suitable alternative to the conventional matching layer, we fabricate 1D linear transducer arrays and packaged the arrays with two types of matching layers: the FPCB matching layer and alumina/epoxy composite matching layer, which is one of the most common acoustic matching layers composed of composite materials. To evaluate the ultrasound transmission characteristics, we measured the electrical and acoustic properties of linear transducer arrays integrated with two types of matching layers. By comparing the ultrasound transmission performance of each transducer array, we confirmed that the FPCB is a promising acoustic matching layer for 1D linear ultrasound transducer arrays. Here we developed a multi-layered composite structure with polyimide and copper layers in the FPCB and applied it as an acoustic matching layer for the first time. The proposed FPCB matching layer offers distinctive advantages over the conventional composite matching layer: (1) the mass-production is possible based on the efficient manufacturing process, and (2) the layer serves the dual functions of acoustic matching and electrical connections for both ground and signals through flexible bending.

## 2. Materials and Methods

### 2.1. Design and Fabrication of the FPCB Matching Layer

To use an FPCB as a matching layer, which is composed of multiple polyimide (PI) layers and copper (Cu) layers, we first determined the composition and thickness of the matching layer. To investigate the optimal composition of PI and Cu layers for acoustic impedance matching, the density (*ρ_FPCB_*), the speed of sound (*c_FPCB_*), and the acoustic impedance of the FPCB matching layer were calculated for various volume fractions of PI and Cu materials (Figure S1). The theoretical density was calculated based on the volume fraction of PI and Cu (1). The theoretical speed of sound was calculated using the theoretical Young’s modulus and the theoretical density which were obtained from the rule of mixture prediction (2) [12].

(1) $$\rho_{FPCB}=\rho_{PI}x_{PI}+\rho_{Cu}x_{Cu}$$

(2) $$c_{FPCB}={\left[(x_{PI}E_{PI}+x_{Cu}E_{Cu})/\rho_{FPCB}\right]}^{1/2}$$

where *ρ_PI_* is the density of PI, *ρ_Cu_* is the density of Cu, *x_PI_* is the volume fraction of PI, and *x_Cu_* is the volume fraction of Cu. *E_PI_* and *E_Cu_* are Young’s modulus of PI and Cu, respectively. The theoretical acoustic impedance was calculated by multiplying the theoretical density with the theoretical speed of sound. Based on the calculation, we chose the composition with 85% PI and 15% Cu, which exhibited the highest ultrasound transmission. The volume fraction of the PI layer could not be further increased since the maximum volume ratio of the PI layer achievable during the fabrication was 85%. With this composition, the ultrasound transmission coefficient of the FPCB matching layer was maximized because its acoustic impedance value was close to 7 MRayl, which is the ideal acoustic impedance for the acoustic matching between the piezoelectric element and water. 

The total thickness of the FPCB matching layer was set to be the quarter-wavelength of the ultrasound transmitted through the FPCB matching layer. The wavelength of the ultrasound was calculated by dividing the theoretical speed of sound in the FPCB matching layer by 1 MHz. With these designs, the FPCB matching layer was fabricated by a typical multilayered FPCB fabrication process followed by board cutting, drilling, dry film imaging, developing, etching, stripping, lamination, surface finish, and packaging [13,14]. The manufacturing process was performed on the copper clad laminate (CCL), a fundamental substrate of the FPCB where the polyimide layer was sandwiched by two copper layers. Specifically, multiple CCL sheets were patterned, stacked up, and laminated to implement a multi-layered composite structure composed of six alternating polyimide and copper layers. First, soft etching was performed on CCL to endow appropriate roughness to the copper layer. A dry film was coated on the pre-treated copper layer and was patterned by UV exposure. The exposed dry film was polymerized, and the unexposed part was removed by developing. The copper layer below the unexposed dry film was also removed via etching, and the remaining dry film on the copper layer was removed by stripping. Next, the patterned CCL sheets were vertically stacked up with alignment, and finally, the FPCB matching layer was fabricated by lamination of the stacked CCL sheets.

### 2.2. Fabrication of the Alumina/Epoxy Matching Layer

For the comparison of the acoustic characteristics with the FPCB matching layer, we fabricated the alumina/epoxy matching layer by mixing alumina (Al_2_O_3_) powder with an epoxy resin (EF-288K42, Sanyu Rec Co., Ltd., Osaka, Japan) [7]. Alumina powder, whose average particle size was 2 μm, was added to the epoxy resin with an 87% weight ratio, and acetone was added into the mixture to dissolve the epoxy resin during mixing. Next, the ball-milling process was performed to disperse alumina powders in the mixture. Then, an imidazole-type curing agent was added to the mixture for the polymerization of the epoxy resin. The mixture was poured into the stainless-steel mold and squeezed until the matching layer reached the desired thickness for quarter-wavelength matching.

### 2.3. Acoustic Property Measurement of Matching Layers

To measure the acoustic properties of the FPCB and the alumina/epoxy matching layers, we calculated the time of flight of the ultrasound passed through matching layers and that of the ultrasound when matching layers are absent (Figure S2) [15]. The FPCB and alumina/epoxy matching layer were placed between the hydrophone and the transducer in the oil tank. A five-cycle sine wave with 200 mV peak-to-peak voltage was generated by a function generator (33220A, Keysight Technologies, Santa Rosa, CA, USA), amplified by a radio frequency amplifier (5312-004 REV. X1, OPHIR RF Inc., Los Angeles, CA, USA), and transmitted through the matching layer by a commercial ultrasound transducer (HAGISONIC, Daejeon, Republic of Korea) [16]. Then, the received signal from a 0.5 mm diameter needle-type hydrophone (NH5000, Precision Acoustics, Dorchester, UK) was amplified and displayed on a digital oscilloscope (DSOX2022A, Keysight Technologies, Santa Rosa, CA, USA).

The speed of sound (c) and acoustic impedance (Z) of the matching layers were calculated with the measured difference in time of flight between transmitted and received signals, the thickness of the matching layer, and reference according to the Formulas (3) and (4) [16].

(3) $$c=\frac{d}{\left(d/c_{o}-\Delta t_{1}+\Delta t_{2}\right)\;}$$

(4)  Z= ρc

where *d* is the thickness of the matching layer, *c_o_* is the speed of sound in oil, ∆t_1_ is the time-of-flight difference between the transmitted and received signal when the matching layer is absent, ∆t_2_ is the time-of-flight difference between the transmitted and received signal when the matching layer is present, and *ρ* is the measured density of the matching layer. The density of the matching layer was calculated by dividing the measured mass of the matching layer into the measured volume of the matching layer, and the thickness of the matching layer was measured using a height gauge (MS-11C, Nikon, Tokyo, Japan). 

The transmission coefficient of an ultrasound wave through the acoustic matching layer was evaluated according to Formula (5) [17].

(5) $$T=\frac{4*\left(Z_{PZT}/Z_{W}\right)}{{\left(Z_{PZT}/Z_{W}+1\right)}^{2}-\left(Z_{PZT}^{2}/Z_{ML}^{2}-1\right)\;*\;(Z_{ML}^{2}/Z_{W}^{2}-1)\;*\sin^{2}kd}$$

where *Z_W_* is the acoustic impedance of water, *Z_PZT_* is the acoustic impedance of the *PZT*, *Z_ML_* is the acoustic impedance of the matching layer, *d* is the thickness of the matching layer, and *k* is the wavenumber.

### 2.4. Fabrication of the 1D Linear Ultrasound Transducer Array with the FPCB Matching Layer

A 1D linear ultrasound transducer array was designed and fabricated to exhibit thickness-mode vibration at 1 MHz, where the thickness of the piezoelectric element was set to 1 mm. The pitch between piezoelectric elements was set to be half-wavelength of the ultrasound in the water so that the grating lobe can be minimized during the ultrasound beam formation [18]. We used the dicing and transfer method for the fabrication. In specific, the fabrication utilized anisotropic conductive film (ACF) bonding of the piezoelectric elements (PZT-5H–3203HD, CTS-Ceramics, Houston, TX, USA) and the flexible interconnect [19,20]. The flexible interconnect consisted of a polyimide/copper composite structure for acoustic matching with a common ground pad, electrical contact pads for piezoelectric elements, and a male-type pin header for the connection with the printed circuit board (PCB). First, the PZT ceramic with 7 mm length and 1 mm thickness was attached to the UV film and diced to 32 elements with a pitch of 1100 μm and space of 35 μm. For the electrical connection of piezoelectric elements and FPCB, ACF (CM-1, H&S Hightech Corp., Daejeon, Korea) was laminated on the electrical contact pads on the FPCB substrate (Figure S3a). Then, the ACF space film was removed from the FPCB substrate (Figure S3b). The pre-bonding of ACF film (CM-1, H&S Hightech Corp., Daejeon, Korea) was performed for 3 s at 120 °C with a pressure of 2.1 MPa to align piezoelectric elements before final bonding (Figure S3c) [21,22]. The final bonding of 32 piezoelectric elements with the FPCB substrate was performed for 15 s at 150 °C and 3.8 MPa to fully cure the ACF and stabilize the bonding of piezoelectric elements and FPCB. After removing UV release film from the piezoelectric elements, another ACF was laminated on a common ground pad on the bottommost part of the FPCB matching layer, which was located on the other side of the flexible interconnect (Figure S3d). Then, the ACF space film on the FPCB matching layer was removed (Figure S3e). Finally, the piezoelectric elements were bonded with a common ground pad of FPCB by folding the other side of the flexible interconnect (Figure S3f,g).

### 2.5. Thickness Uniformity Measurement of 1D Linear Ultrasound Transducer Arrays

Thickness uniformity of the 1D linear ultrasound transducer array without an acoustic matching layer, with the FPCB matching layer, and with the alumina/epoxy matching layer was measured with a height gauge (MS-11C, Nikon, Japan). The matching layer with a size of 26 mm × 10 mm was equally divided into four parts in the x direction and three parts in the y-direction (i.e., a total of 12 points). The thickness uniformity of the matching layer was estimated by the difference of the thickness at each point to the measured average thickness of the matching layer at that point.

### 2.6. Electrical Characterization of 1D Linear Ultrasound Transducer Arrays

An electrical input impedance of 1D linear ultrasound transducer array without an acoustic matching layer, with the FPCB matching layer, and with the alumina/epoxy matching layer were measured using an impedance analyzer (E4990A, Keysight Technologies, Santa Rosa, CA, USA) and probe station. To operate 32 piezoelectric elements of 1D linear ultrasound transducer arrays at the same time, an additional custom-designed PCB was prepared. A male-type pin header on the FPCB substrate was inserted into the female-type pin header of the PCB, and the PCB was connected to the impedance analyzer via BNC cable to apply the AC bias to 1D linear ultrasound transducer arrays. The input amplitude and phase of 1D linear ultrasound transducer arrays were measured, and the resonance characteristics of each device were investigated.

### 2.7. Acoustic Characterization of 1D Linear Ultrasound Transducer Arrays

The maximum output pressure, bandwidth, and ultrasound beam profile of the 1D linear ultrasound transducer array were measured by the through-transmission method in a cylindrical glass beaker filled with soybean oil. A 0.5 mm diameter needle-type hydrophone (NH0500, Precision Acoustics, UK) was fixed to a motorized stage (Sciencetown Inc., Incheon, Korea) which was programmed to allow scanning on the micrometer scale. A five-cycle sine wave with 200 mV peak-to-peak voltage was generated by a function generator (33220A, Keysight Technologies, CA, USA), amplified by a radio frequency amplifier (5312-004 REV. X1, OPHIR RF Inc., LA, USA), and applied to 1D linear ultrasound transducer array which was fixed to a clamp and placed in front of the hydrophone. The transmitted ultrasound signal was received by a 0.5 mm diameter needle-type hydrophone (NH5000, Precision Acoustics, UK), amplified, and displayed on a digital oscilloscope (DSOX2022A, Keysight Technologies, CA, USA). To obtain the ultrasound beam profile of a 1D linear ultrasound transducer array, a single vertical scan was performed in the x-direction to find the point where the beam intensity is maximum. An additional scan was performed across the horizontal plane (26 × 16 mm^2^) in the yz direction with a step resolution of 300 µm while maintaining the x position [16]. The maximum output pressure of the 1D linear ultrasound transducer array was calculated by dividing the measured peak-to-peak voltage of the received signal at the point of maximum intensity by the previously calibrated sensitivity of the hydrophone. A pulse wave with one cycle and 100 ns pulse width was transmitted via a 1D linear ultrasound transducer array for the bandwidth measurement. Fast Fourier transform (FFT) was performed on the received signal to obtain the normalized amplitude of the signal at the frequency domain.

## 3. Results

### 3.1. Acoustic Properties of the FPCB Matching Layer

One of the advantages of applying an FPCB as an acoustic matching layer is that the acoustic characteristics can be readily controlled by adopting different volume fractions and adjusting the position of polyimide layers and copper layers in the FPCB. In this study, we designed the FPCB matching layer with the volume fraction of polyimide and copper to 85% and 15%, respectively. Considering that the density of the composite material consisting of two materials linearly depends on the density and the volume fraction of each material [23], we minimized the volume fraction of the copper layer to decrease the density and thus the acoustic impedance. To investigate the acoustic properties of matching layers and determine the suitability of applying the FPCB as an acoustic matching layer based on the quarter-wavelength matching theory, we measured the density and speed of sound, calculated acoustic impedance, and compared the transmission coefficient of the FPCB and alumina/epoxy matching layers for varying frequencies from 1 MHz to 5 MHz.

**Figure 2:**
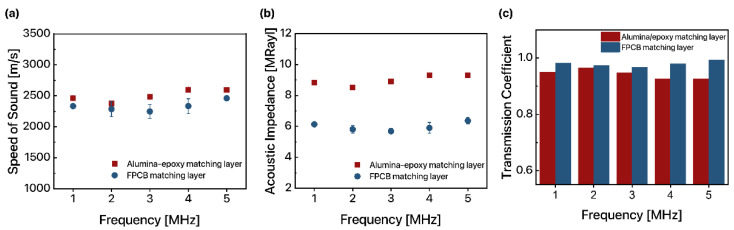
Acoustic properties of the alumina/epoxy matching layer and FPCB matching layer: (**a**) Speed of sound, (**b**) acoustic impedance, and (**c**) transmission coefficient of the ultrasound through the matching layer at different frequencies (*n =* 4).

The measured density of the alumina/epoxy matching layer and FPCB matching layer was 3585 kg/m^3^ and 2630 kg/m^3^, respectively. The average speed of sound in the alumina/epoxy matching layer was 2462 m/s, 2374 m/s, 2482 m/s, 2599 m/s, and 2596 m/s for 1 MHz, 2 MHz, 3 MHz, 4 MHz, and 5 MHz, respectively (3) (Figure 2a). The average speed of sound in the FPCB matching layer was 2333 m/s, 2286 m/s, 2248 m/s, 2334 m/s, and 2460 m/s for 1 MHz, 2 MHz, 3 MHz, 4 MHz, and 5 MHz, respectively. The acoustic impedance of the alumina/epoxy matching layer was 8.83 MRayl, 8.51 MRayl, 8.9 MRayl, 9.32 MRayl, and 9.31 MRayl for 1 MHz, 2 MHz, 3 MHz, 4 MHz, and 5 MHz, respectively (4) (Figure 2b). The acoustic impedance of the FPCB matching layer was 6.14 MRayl, 5.81 MRayl, 5.69 MRayl, 5.91 MRayl, and 6.36 MRayl for 1 MHz, 2 MHz, 3 MHz, 4 MHz, and 5 MHz, respectively. Finally, the transmission coefficient was calculated by assuming the thickness of the matching layer was quarter-wavelength so that we could solely compare the effect of acoustic impedance of the material (5). The thickness (*d*) and corresponding wavenumber (*k*) of the alumina/epoxy and FPCB matching layers are summarized in Table 1. The transmission coefficient of the alumina/epoxy matching layer was 0.95, 0.965, 0.947, 0.925, and 0.926 for 1 MHz, 2 MHz, 3 MHz, 4 MHz, and 5 MHz, respectively (Figure 2c). The transmission coefficient of the FPCB matching layer was 0.981, 0.973, 0.967, 0.979, and 0.993 for 1 MHz, 2 MHz, 3 MHz, 4 MHz, and 5 MHz, respectively. Assuming the thickness of the matching layer is quarter-wavelength, the ultrasound transmission coefficient is maximized when the acoustic impedance of the matching layer is the geometric mean of the acoustic impedance of two surrounding media around the matching layer [3]. Since the acoustic impedance of the FPCB matching layer was closer to the geometric mean of the acoustic impedance of PZT and water (7.04 MRayl) than that of alumina/epoxy, ultrasound transmission was higher when the FPCB was applied as an acoustic matching layer compared to when alumina/epoxy was applied. As a consequence, the ultrasound transmission through the FPCB matching layer was higher compared to that through the alumina/epoxy matching layer for all ultrasound frequencies tested, which confirms that FPCB is sufficient to be applied as an acoustic matching layer.

### 3.2. Physical Characteristics of 1D Linear Ultrasound Transducer Arrays

**Figure 3:**
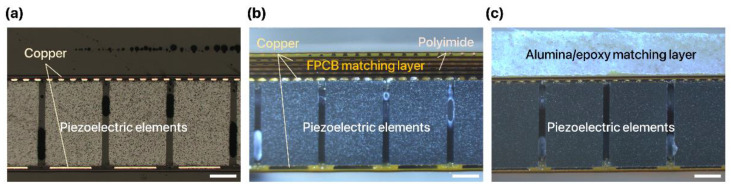
Cross-sectional optical images of 1D linear ultrasound transducer arrays (**a**) without acoustic matching layer, (**b**) with the FPCB matching layer, and (**c**) with the alumina/epoxy matching layer (scale bar: 200 µm).

We successfully fabricated a 1D linear ultrasound transducer array with an FPCB matching layer. Due to the flexibility of the substrate and the robustness of the ACF bonding, the FPCB was readily folded and integrated with the 1D linear PZT array consisting of 32 elements. With this configuration, the FPCB not only serves as an acoustic matching layer between the piezoelectric element and the surrounding medium but also as a ground for the electrical connection between the piezoelectric elements and the folded substrate. For the 1D linear ultrasound transducer array without a matching layer, only a single-layer FPCB was applied and bonded to the piezoelectric elements for the electrical connection (Figure 3a). The thickness of the FPCB matching layer and the alumina/epoxy matching layer was designed to be the quarter-wavelength for the maximization of the ultrasound transmission. The measured thickness of the FPCB matching layer and alumina/epoxy matching layer was 478.4 μm and 537.03 μm, respectively.

**Figure 4:**
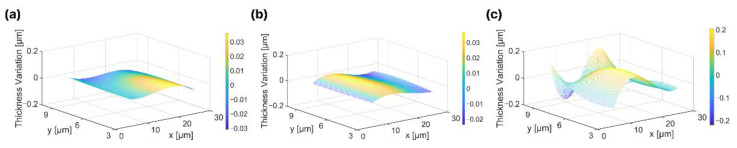
Thickness variation of 1D linear ultrasound transducer arrays (**a**) without acoustic matching layer, (**b**) with the FPCB matching layer, and (**c**) with the alumina/epoxy matching layer.

The fabricated FPCB acoustic matching layer clearly exhibited the composite structure where six copper and polyimide layers were sequentially stacked on the substrate via an adhesive (Figure 3b). Every copper pattern in the matching layer exhibited a highly consistent and regular configuration. However, the alumina/epoxy matching layer exhibited a highly random and nonuniform arrangement of particles inside the layer (Figure 3c). Furthermore, we measured the thickness variation of the matching layers after completing the fabrication to investigate the uniformity of the matching layer. Uniformity of the matching layer is important as it affects the evenness and the predictability of the ultrasound transmission characteristics [24]. The FPCB matching layer exhibited a much lower variation of the thickness across the surface than the alumina/epoxy matching layer. The average thickness variation was 0.015 μm, 0.057 μm, and 0.099 μm for the 1D linear ultrasound transducer arrays without a matching layer (Figure 4a), with the FPCB matching layer (Figure 4b), and with the alumina/epoxy matching layer (Figure 4c), respectively. While the thickness measurement points of the samples without a matching layer and the 6-layer FPCB matching layer were distributed near the average thickness plane (z = 0), measurement points of the alumina/epoxy matching layer largely deviated from the average thickness plane. The nonuniformity of the alumina/epoxy matching layer is attributed to the fabrication process where the composite mixture is poured into the epoxy mold, cured, and squeezed to the desired thickness [11]. Therefore, it is more advantageous to apply the FPCB as an acoustic matching layer as we can manufacture a highly uniform layer with a simple PCB fabrication process. 

### 3.3. Electrical Characterization of 1D Linear Ultrasound Transducer Arrays

**Figure 5:**
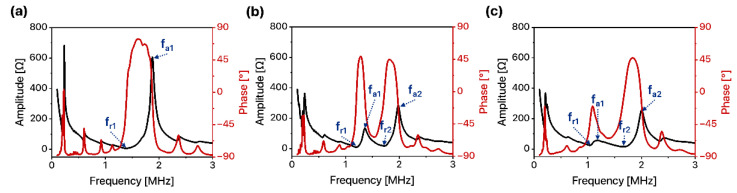
Electrical input impedance of 1D linear ultrasound transducer arrays (**a**) without matching layer, (**b**) with the FPCB matching layer, and (**c**) with the alumina/epoxy matching layer.

To analyze resonance characteristics, we measured the electrical input impedance of the 1D linear ultrasound transducer arrays with three different types of matching layers: no acoustic matching layer, the FPCB matching layer, and the alumina/epoxy matching layer. Since all transducer arrays adopted PZT-5H as a piezoelectric element whose resonance frequency range is 1–2 MHz during thickness mode vibration, a resonance peak near 1–2 MHz was analyzed in detail. For the case of a 1D linear ultrasound transducer array without any acoustic matching layer, a single resonance peak was observed. The resonance frequency (f_r_) and anti-resonance frequency (f_a_) of the device were 1.4 MHz and 1.88 MHz, respectively (Figure 5a).

However, the resonance characteristics of the 1D linear ultrasound transducer array changed with the application of the acoustic matching layer. Multiple resonance peaks were observed as acoustic matching layers were introduced to the 1D linear ultrasound transducer arrays [25]. A 1D linear ultrasound transducer array with an FPCB matching layer exhibited two strong resonances (Figure 5b). The first resonance frequency (f_r1_) and anti-resonance frequency (f_a1_) were 1.14 MHz and 1.32 MHz, respectively. The second resonance frequency (f_r2_) and anti-resonance frequency (f_a2_) were 1.66 MHz and 1.95 MHz, respectively. A 1D linear ultrasound transducer array with the alumina/epoxy matching layer also exhibited two main resonance peaks, but the amplitude of the first resonance peak was decreased and the distance between the first and the second peak was larger (Figure 5c). The first resonance frequency (f_r1_) and anti-resonance frequency (f_a1_) were 1.04 MHz and 1.19 MHz, respectively. The second resonance frequency (f_r2_) and anti-resonance frequency (f_a2_) were 1.66 MHz and 2.01 MHz, respectively. The difference in resonance characteristics of the two devices originated from the acoustic properties of two matching layers. The larger density and higher acoustic impedance of the alumina/epoxy matching layer led to a larger load mass on the transducer, which resulted in a lower amplitude peak and a larger distance between resonance peaks [26]. The main resonance frequency of the device was determined where the impedance amplitude was the minimum: 1.4 MHz for a 1D linear ultrasound transducer array with the FPCB matching layer and 1.66 MHz for a 1D linear ultrasound transducer array with the alumina/epoxy matching layer.

### 3.4. Acoustic Properties of 1D Linear Ultrasound Transducer Arrays

#### 3.4.1. Output Pressure and Bandwidth

**Figure 6:**
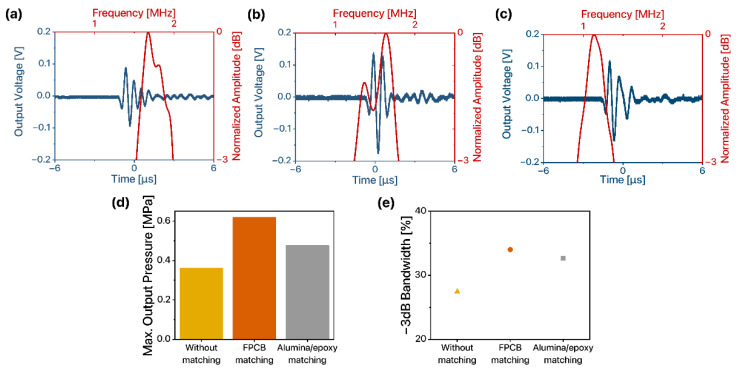
Acoustic characteristics of 1D linear ultrasound transducer arrays (**a**) without matching layer, (**b**) with the FPCB matching layer, and (**c**) with the alumina/epoxy matching layer. (**d**) Maximum output pressure. (**e**) −3 dB bandwidth.

We compared the ultrasound transmission performance of the 1D linear ultrasound transducer arrays with different types of matching layers by measuring output pressure and fractional bandwidth. For the interface consisting of the same materials, the reflection coefficient, the ratio of the amplitude of the reflected ultrasound to that of the incident ultrasound, remains the same [27]. As a result, for the same interface, it is important for ultrasound transducers to emit large output pressure since it leads to a stronger echo signal and easier detection of the reflected signal. Furthermore, it is desirable for 1D linear ultrasound transducer arrays to exhibit larger fractional bandwidth since it contributes to the higher resolution and quality of ultrasound imaging [28,29]. To compare the maximum output performance of 1D linear ultrasound transducer arrays, we applied an AC signal with a frequency equivalent to the resonant frequency of each 1D linear ultrasound transducer array determined from the electrical characterization. We measured the peak-to-peak voltage of the output signal received by the hydrophone and normalized the amplitude of the signal in the frequency domain to estimate the −3 dB bandwidth (Figure 6a–c). The peak-to-peak voltage of the output signal was 0.183 V, 0.314 V, and 0.249 V for 1D linear ultrasound transducer without the matching layer, with the FPCB matching layer, and with the alumina/epoxy matching layer, respectively. Two dominant peaks were observed in the normalized amplitude of the 1D linear ultrasound transducer array with the FPCB matching layer due to the multi-layered composite structure of the FPCB matching layer. The use of multiple acoustic matching layers changes the ultrasound transmission performance of the transducers, and the frequency range of the resonance varies with the type of material applied for the acoustic matching layer [25,30]. Due to multiple copper layers and polyimide layers in the FPCB matching layer, the vibration modes of the piezoelectric elements and thus, ultrasound transmission performance of the 1D linear ultrasound transducer array was altered. Therefore, unlike the 1D linear ultrasound transducer array without a matching layer (i.e., when a single layer FPCB was applied) which exhibited a single peak, two resonant peaks were observed when the FPCB matching layer was used.

Without any acoustic matching layers applied, the maximum output pressure of the 1D linear ultrasound transducer array was 0.39 MPa (Figure 6d). The maximum output pressure was enhanced when coupled to acoustic matching layers. In specific, the output pressure was maximized when using the FPCB matching layer. The maximum output pressure of the 1D linear ultrasound transducer arrays with the FPCB and the alumina/epoxy matching layers was 0.61 MPa and 0.48 MPa, respectively. The −3 dB bandwidth of the 1D linear ultrasound transducer array was also the largest for the FPCB matching layer case (Figure 6e). The −3 dB bandwidth of the 1D linear ultrasound transducer array without an acoustic matching layer was 27.42%, while that coupled to the FPCB matching and alumina/epoxy matching layers was 34.01% and 32.65%, respectively. The 1D linear ultrasound transducer array with the FPCB matching layer exhibited better transmission performance than that with the alumina/epoxy matching layer. These results are attributed to the acoustic properties of the FPCB matching layer which exhibited an acoustic impedance closer to 7 MRayl and high thickness uniformity. The ultrasound transmission performance of the 1D linear ultrasound transducer array can be further enhanced by applying highly attenuating material as a backing layer at the backside of the piezoelectric element [31,32,33]. The effect of the type and the structure of the backing layer on the performance of the 1D linear ultrasound transducer array should be further studied.

#### 3.4.2. Ultrasound Beam Profile

**Figure 7:**
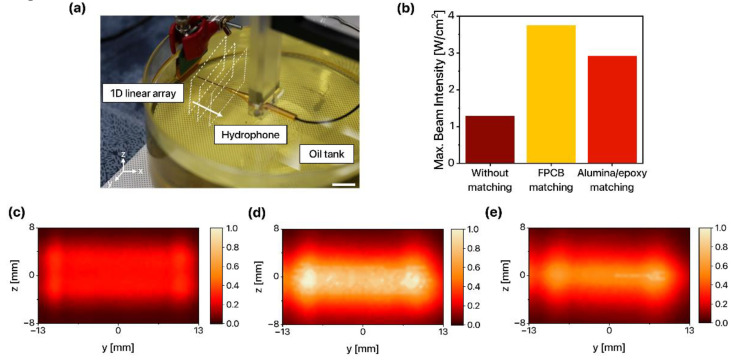
(**a**) Photo of the experimental setup for ultrasound beam profile measurement (scale bar: 1 cm). (**b**) Maximum ultrasound beam intensity and transversal ultrasound beam profiles of 1D linear ultrasound transducer arrays (**c**) without matching layer, (**d**) with the FPCB matching layer, and (**e**) with the alumina/epoxy matching layer.

The transversal ultrasound beam profile of the 1D linear ultrasound transducer arrays at the plane of maximum intensity was measured to assess the shape and uniformity of the ultrasound beam and to compare the maximum beam intensity (Figure 7a). The maximum output beam intensity of the 1D linear ultrasound transducer array without an acoustic matching layer was 1.28 W/cm^2^. When the FPCB and alumina/epoxy acoustic matching layers were applied to the 1D linear ultrasound transducer arrays, the maximum output beam intensity was enhanced (3.75 W/cm^2^ and 2.92 W/cm^2^, respectively) (Figure 7b).

Furthermore, the transversal ultrasound beam profile was normalized from 0 to 1 with the maximum beam intensity of all and was visualized as a heatmap based on the beam intensity of the 1D linear ultrasound transducer array with the FPCB matching layer. While the ultrasound beam of the 1D linear ultrasound transducer array without a matching layer (Figure 7c) was not clearly distinguishable from the background due to the low intensity, the ultrasound beam of the 1D linear ultrasound transducer arrays with the FPCB matching layer (Figure 7d) and the alumina/epoxy matching layer (Figure 7e) clearly exhibited the shape of the 1D linear ultrasound transducer arrays. The ultrasound beam of the 1D linear ultrasound transducer array integrated with the FPCB matching layer exhibited the brightest color and the largest contrast. Not only the maximum beam intensity but average beam intensity was the largest for the FPCB case, which implies that the ultrasound energy transmission was maximized. In conclusion, the FPCB enhances the transmission performance of 1D linear ultrasound transducer arrays and therefore is a promising candidate for an acoustic matching layer for 1D linear ultrasound transducer arrays.

## 4. Conclusions

We proposed and demonstrated an FPCB as a novel acoustic matching layer for 1D linear ultrasound transducer arrays. Due to the flexibility of the substrate and ACF bonding, the FPCB could be folded and bonded with the other side of the substrate for consistent and convenient packaging. In this scheme, the FPCB not only serves as an acoustic matching layer between the piezoelectric element and the surrounding medium but also as a ground for the electrical connection between the transducer array elements and the folded substrate. With a composite structure consisting of multiple polyimide layers and copper layers, the FPCB exhibited an acoustic impedance close to the geometric mean of the acoustic impedance of the piezoelectric element and that of the surrounding medium, which enhanced the ultrasound energy transmission through the layer. A 1D linear ultrasound transducer array with the FPCB matching layer exhibited relatively high output pressure, a wide frequency bandwidth, and a high ultrasound beam intensity. Thus, the proposed FPCB is a promising acoustic matching layer for 1D linear ultrasound transducer arrays.

## Figures and Tables

**Table 1:** Summary of the calculated thickness (*d*) and the corresponding wavenumber (*k*) used to estimate transmission coefficients of the alumina/epoxy and FPCB matching layers.

Matching Layer	Frequency [MHz]	Thickness (*d*) [μm]	Wavenumber (*k*) [cm^−1^]	Transmission Coefficient
Alumina/epoxy	1	620	25.5	0.95
	2	297	52.9	0.965
	3	207	75.9	0.947
	4	162	96.7	0.925
	5	130	120.9	0.926
FPCB	1	583	26.9	0.981
	2	286	55.1	0.973
	3	187	84	0.967
	4	146	107.9	0.979
	5	123	127.6	0.993

## Data Availability

Not applicable.

## Supplementary Materials

The following are available online at https://www.mdpi.com/article/10.3390/s22155557/s1, Figure S1: Theoretical acoustic properties of the FPCB matching layer composed of different volume fractions of polyimide and copper: (a) density and speed of sound, (b) acoustic impedance, and (c) transmission coefficient, Figure S2: Photo of the experimental setup for acoustic characterization of FPCB matching layer, Figure S3: Optical images of the packaging steps: (a) ACF lamination on the FPCB substrate (scale bar: 2 cm), (b) removal of ACF space film from the FPCB substrate (scale bar: 7 mm), (c) ACF pre-bonding and delamination of the UV film from piezoelectric elements (scale bar: 1 cm), (d) ACF lamination on the FPCB matching layer (scale bar: 1 cm), (e) removal of ACF space film from the FPCB matching layer (scale bar: 1 cm), (f) final ACF bonding of the FPCB matching layer and piezoelectric elements (scale bar: 7 mm), and (g) successfully packaged devices (scale bar: 2 cm).
