# Supplementary material for: FPCB as an Acoustic Matching Layer for 1D Linear Ultrasound Transducer Arrays
Source: Sensors (Basel). 2022 Jul 25;22(15):5557. doi: 10.3390/s22155557 (PMC9332256; doi:10.3390/s22155557)
Supplement: Supplementary file 1 [file sensors-22-05557-s001.zip › sensors-1813865-supplementary.pdf]

Supplementary Material

FPCB as an Acoustic Matching Layer for 1D Linear Ultrasound Transducer Arrays

Taemin Lee [1,†], Joontaek Jung [2,†], Sang-Mok Lee [1], Jongcheol Park [2], Jae-Hyeong Park [3], Kyung-Wook Paik [4], and Hyunjoo J. Lee [1,*]

[1]*School of Electrical Engineering, Korea Advanced Institute of Science and Technology, Daejeon 34141, Republic of Korea; leetm@kaist.ac.kr (T.L.); mock0920@kaist.ac.kr (S.-M.L.)*

[2]*Office of Nano Convergence Technology, National NanoFab Center, Daejeon 34141, Republic of Korea; jjt@nnfc.re.kr (J.J.); jcpark@nnfc.re.kr (J.P.)*

[3]*Samsung Foundry, Samsung Electronics co., Ltd; jh3375.park@samsung.com (J.-H.P.)*

[4]*Department of Materials Science and Engineering, Korea Advanced Institute of Science and Technology, Daejeon 34141, Republic of Korea; kwpaik@kaist.ac.kr (K.-W.P.)*

**Correspondence: hyunjoo.lee@kaist.ac.kr; Tel.: +82-42-350-7536 (H.J.L.)*

[†]*These authors contributed equally to this work.*

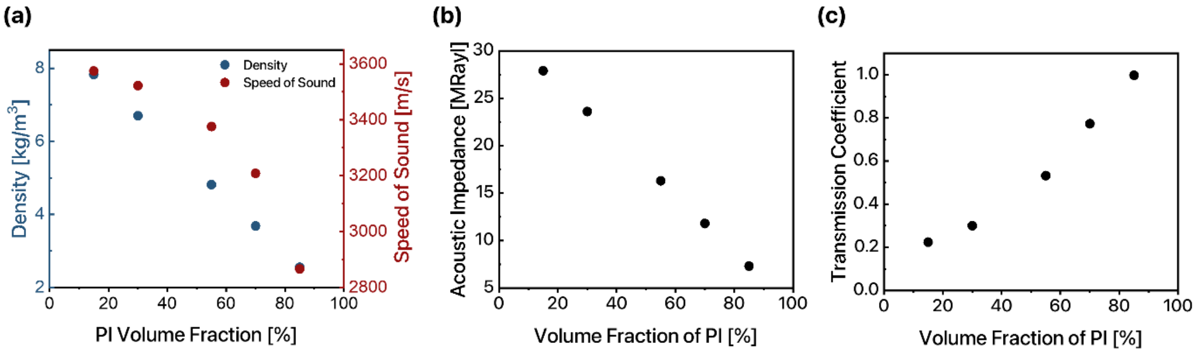

Figure S1. Theoretical acoustic properties of the FPCB matching layer composed of different volume fractions of polyimide and copper: (a) density and speed of sound, (b) acoustic impedance, and (c) transmission coefficient.

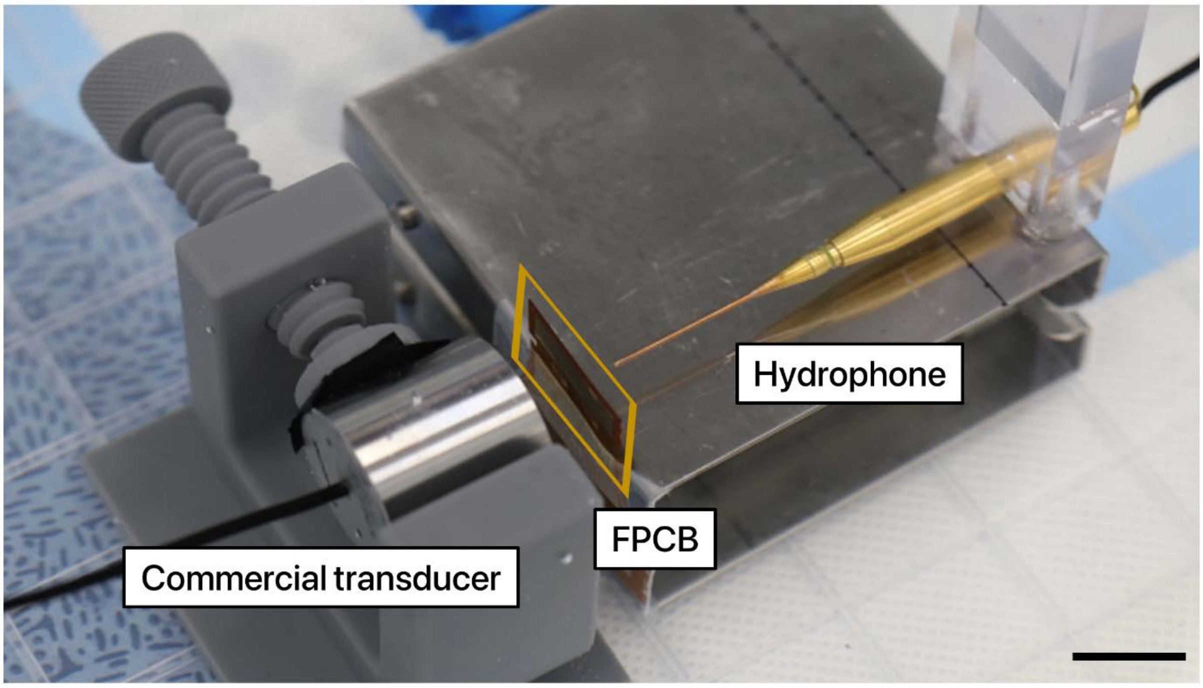

Figure S2. Photo of the experimental setup for acoustic characteriztion of the FPCB matching layer (scale bar: 2 cm).

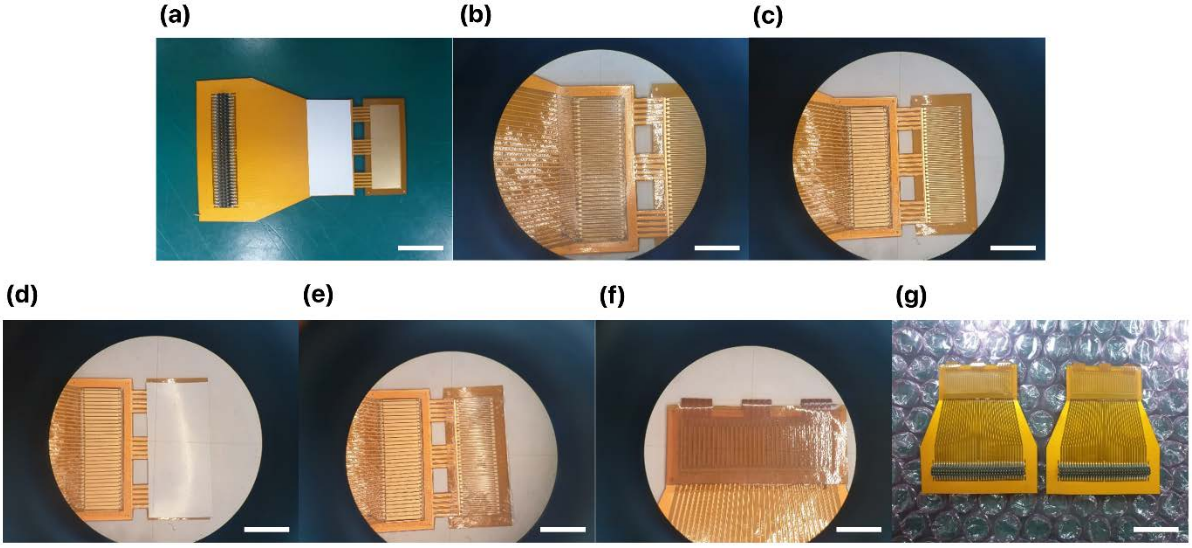

Figure S3. Optical images of the packaging steps: (a) ACF lamination on the FPCB substrate (scale bar: 2 cm), (b) removal of ACF space film from the FPCB substrate (scale bar: 7 mm), (c) ACF pre-bonding and delamination of the UV film from piezoelectric elements (scale bar: 1 cm), (d) ACF lamination on the FPCB matching layer (scale bar: 1 cm), (e) removal of ACF space film from the FPCB matching layer (scale bar: 1 cm), (f) final ACF bonding of the FPCB matching layer and piezoelectric elements (scale bar: 7 mm), and (g) successfully packaged devices (scale bar: 2 cm).
